# POLICY SERIES: HEALTH AND AGING POLICY FELLOWS: CONNECTING EXPERIENCE TO POLICY

**DOI:** 10.1093/geroni/igae098.0196

**Published:** 2024-12-31

**Authors:** Maureen Henry, Pi Ju Liu

**Affiliations:** Chair; Discussant

## Abstract

For health and aging policy to be effective, it must link an understanding of how systems impact individuals to an understanding of how policy influences the systems. Systems such as health care and social service delivery, family caregiving, lifelong learning, zoning laws, and the housing market influence older people and their families. Policy can make these systems better - or worse - for supporting the health and wellbeing for older people. The Health and Aging Policy Fellows program provides experts with real-life experience the opportunity to learn how policy works so they can better support older people. This symposium will describe how current fellows are addressing federal policy within congress and administrative agencies, state policy through a non-profit organization comprising state aging officials, and across government through a non-profit organization focused on caregiving policy. The 2024 reauthorization of the Older Americans Act is an area of focus across all settings. Fellows are also working on care and payment models in primary care and geriatrics; assisted living facilities; direct care workforce; family caregiving policy and research agendas; elder justice, including Adult Protective Services, the Long-Term Care Ombudsman, guardianship). During the symposium, fellows will discuss these and related topics the context of their fellowship experiences.

